# Targeting Neurological Disorders with Stilbenes: Bridging the Preclinical-Clinical Gap

**DOI:** 10.7150/ijbs.102032

**Published:** 2024-10-07

**Authors:** Md. Zamshed Alam Begh, Jishan Khan, Mehrukh Zehravi, Sherouk Hussein Sweilam, A. Dinesh Raja, A. Muthukumar, M Akiful Haque, Nihar Ranjan Kar, Laliteshwar Pratap Singh, B. Dharani Priya, Mohammed Ali Alshehri, Irfan Ahmad, Sojin Kang, Seungjoon Moon, Moon Nyeo Park, Talha Bin Emran, Bonglee Kim

**Affiliations:** 1 Department of Pharmacy, Faculty of Allied Health Sciences, Daffodil International University, Dhaka 1207, Bangladesh.; 2 Department of Pharmacy, International Islamic University Chittagong, Kumira, Chittagong 4318, Bangladesh.; 3 Department of Clinical Pharmacy, College of Dentistry & Pharmacy, Buraydah Private Colleges, Buraydah 51418, Saudi Arabia.; 4 Department of Pharmacognosy, College of Pharmacy, Prince Sattam Bin Abdulaziz University, Al-Kharj 11942, Saudi Arabia.; 5 Department of Pharmacognosy, Faculty of Pharmacy, Egyptian Russian University, Cairo-Suez Road, Badr City, Cairo 11829, Egypt.; 6 Department of Pharmaceutics, KMCH College of Pharmacy, Coimbatore, Tamil Nadu 641048, India.; 7 Department of Pharmacology, The Oxford College of Pharmacy, Bengaluru, Karnataka 560068, India.; 8 Department of Pharmaceutical Analysis, School of Pharmacy, Anurag University, Hyderabad, India.; 9 Centurion University of Technology and Management, Gopalpur, Balasore 756044, Odisha, India.; 10 Department of Pharmaceutical Chemistry, Narayan Institute of Pharmacy, Gopal Narayan Singh University, Sasaram 821305, India.; 11 Department of Biology, Faculty of Science, University of Tabuk, Tabuk 71491, Saudi Arabia.; 12 Department of Clinical Laboratory Sciences, College of Applied Medical Science, King Khalid University, Abha 61421, Saudi Arabia.; 13 Department of Pathology, College of Korean Medicine, Kyung Hee University, 26 Kyungheedae-ro, Dongdaemun-gu, Seoul, 02453, Republic of Korea.; 14 Department of Pathology and Laboratory Medicine, Warren Alpert Medical School, Brown University, Providence, RI 02912, USA.

**Keywords:** Stilbenes, Chemistry, Neurological disorders, Preclinical studies, Clinical studies

## Abstract

Neurological disorders (NDs) encompass a range of debilitating conditions that affect the nervous system, including prevalent illnesses such as Alzheimer's disease, Parkinson's disease, and ischemic stroke. Despite significant ongoing studies, effective therapeutic strategies to halt or slow down the progression of these illnesses are still lacking. Stilbenes, a class of natural polyphenols, have shown potential as candidates for therapeutic strategies due to their capacity to protect the nervous system. Preclinical studies have provided strong evidence that stilbenes can regulate many cellular pathways implicated in neurodegeneration, with resveratrol being a well-studied compound that has shown the ability to reduce oxidative damage, promote neurogenesis, and enhance mitochondrial function - crucial for maintaining brain health. In preclinical animal models, initial research has also shown promise in additional substances such as piceatannol and pterostilbene. Furthermore, clinical studies have explored the therapeutic benefits of stilbenes in NDs. Despite promising results in preclinical research, the use of stilbenes in clinical trials is currently limited, with most studies focusing on resveratrol. Although several clinical studies have demonstrated the beneficial impact of resveratrol supplementation on brain health and degenerative consequences, other investigations have yielded ambiguous findings, underscoring the urgent need for more comprehensive and precisely planned clinical research. This study delves into the potential benefits of stilbenes as neuroprotective agents for NDs. It emphasizes the need for more clinical research to enhance our understanding of their therapeutic effectiveness in specific patient groups.

## 1. Introduction

Neurological disorders (NDs) encompass a diverse group of debilitating conditions that affect the nervous system, including the brain, spinal cord, nerves, and muscles. These disorders are characterized by a progressive decline in cognitive function, motor skills, and sensory perception, significantly impacting the quality of life for millions of individuals worldwide 1. With an aging population and increasing environmental pressures, the burden of NDs will rise dramatically in the coming decades 2. Unfortunately, current treatment options for many NDs are limited. While some medications can manage symptoms and slow disease progression, there is a critical lack of therapies that can halt or reverse the underlying neurodegenerative processes 3. This necessitates the exploration of novel therapeutic avenues, and natural products, with their inherent biological activity and often minimal side effects, have emerged as promising candidates 4. Among these natural products, stilbenes, a class of polyphenolic compounds, have garnered significant interest for their potential neuroprotective properties 5. Resveratrol, the most well-known stilbene, is abundant in red wine, grapes, peanuts, and certain berries. Its ability to activate sirtuins, a family of enzymes known to regulate cellular stress responses and longevity pathways, has placed it at the forefront of research investigating its potential benefits against aging-related diseases, including neurodegeneration 6,7. However, stilbenes extend far beyond resveratrol. Numerous stilbene derivatives are occurring naturally or synthesized in the laboratory, each with its unique chemical structure and potentially distinct biological effects. This structural diversity presents a vast landscape for exploring stilbenes with enhanced potency, improved bioavailability, and targeted therapeutic actions against specific NDs 8,9.

Stilbenes are being explored as potential neuroprotective agents due to their multifaceted approach to addressing the complex cellular and molecular events contributing to neurodegeneration 10. They have been found to exert their beneficial effects through various mechanisms, including antioxidant activity, anti-inflammatory effects, enhancement of neurotrophic factors, protein aggregation inhibition, and modulation of autophagy. These properties protect neurons from oxidative stress (OS), often driven by OS 11. Stilbenes have also been shown to modulate inflammatory pathways, potentially mitigating neuroinflammation and promoting neuronal survival. They may also enhance neurotrophic factors essential for neuronal growth and function 11,12. They have also shown promise in preventing protein misfolding and aggregation, potentially hindering the progression of neurodegenerative disorders 13. Despite the promising preclinical evidence, significant research is needed to translate these benefits into clinical applications fully. Challenges include optimizing the delivery and bioavailability of stilbenes in the brain, ensuring their safety for long-term use, and establishing their efficacy in well-designed clinical trials 14. The growing body of research on stilbenes offers hope in the labyrinth of NDs. With their diverse bioactivities and promising preclinical data, stilbenes hold immense potential for becoming a valuable weapon in the fight to protect and preserve neurological health 15. Transitioning from preclinical evidence to clinical applications is crucial for developing stilbenes as potential therapeutic candidates for NDs. This involves understanding their mechanisms of action, pharmacokinetic profiles, safety profiles, and efficacy in relevant models and translating these findings into robust clinical trials to evaluate their therapeutic potential 15.

This review evaluates stilbenes' potential as treatments for NDs, focusing on their transition from preclinical evidence to clinical applications. It examines their pharmacological properties, mechanisms of action, preclinical studies, and clinical trials to understand their potential.

## 2. Stilbenes: an overview

**Figure 1:**
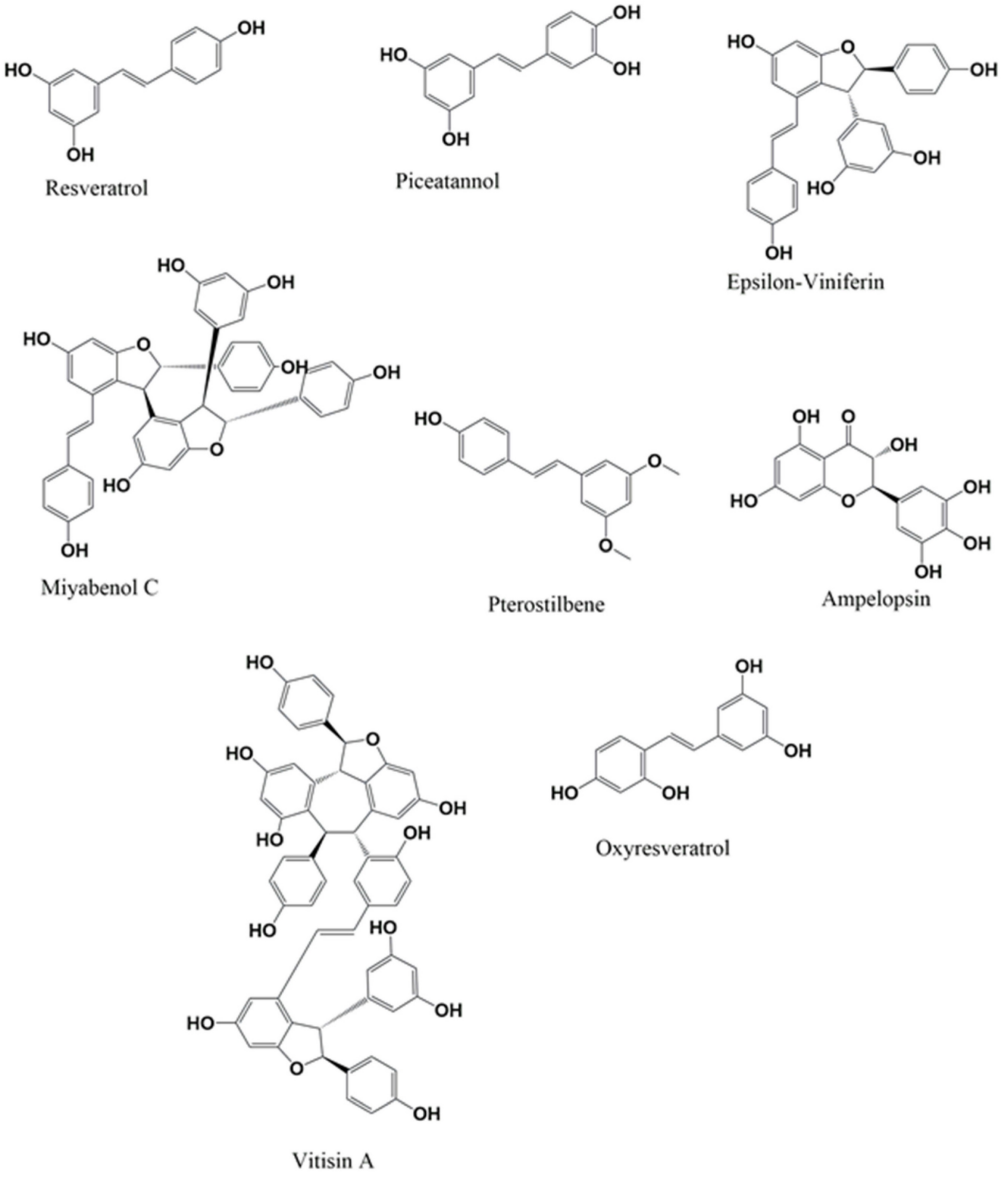
Chemical structure of stilbenes.

Stilbenes, a group of polyphenolic compounds, have gained significant attention in recent years due to their potential therapeutic applications, particularly in treating NDs. The structure-activity relationship (SAR) of stilbenes is attributed to the pharmacological actions of stilbenes 16. The review will particularly emphasize the neuroprotective effects of stilbenes. Suppose researchers possess a strong understanding of the SAR of stilbenes. In that case, they may develop and produce novel compounds that exhibit improved efficacy and specificity in treating NDs 17. The defining characteristic of stilbenes (Figure 1), as opposed to other compounds, is the core 1,2-diphenylethylene backbone, consisting of two phenyl rings joined by an ethylene bridge. Trans-resveratrol, a stilbene molecule, is well-recognized by most people. It is often found in red grapes and wine in significant amounts 17. Pterostilbene, pinosylvin, and resveratrol dimers such as ε-vinifera and δ-vinifera are naturally occurring stilbenes distinct from other stilbenes 18. To achieve medical treatment objectives, synthetic derivatives of stilbene have been developed, expanding the chemical diversity within this class of compounds 19.

The SAR represents the pharmacological characteristics of stilbenes, namely their ability to protect the nervous system. The main structural elements are hydroxyl groups linked to the stilbene backbone, which enhance the antioxidant activity and anti-inflammatory properties 20. For instance, piceatannol, which has three hydroxyl groups, demonstrates more antioxidant activity than resveratrol, which only has two hydroxyl groups 21. The introduction of methoxy substitutions at specific positions on the phenyl rings may modify the level of lipid solubility and metabolic stability, hence impacting the pharmacokinetic properties of the molecules. Pterostilbene, a naturally occurring chemical, is a dimethylated derivative of resveratrol. It has a more remarkable ability to be absorbed by the body and taken up by cells than in its original form. This may potentially enhance its efficacy in safeguarding the brain 22. The stereochemistry of the double bond in stilbene compounds substantially influences their biological activity 23. Trans-stilbenes, characterized by a double bond in a trans conformation, often demonstrate superior bioactivity compared to their cis counterparts. Trans-resveratrol has neuroprotective effects via many mechanisms, such as activating sirtuins, decreasing amyloid-beta (Aβ) aggregation, and altering signaling pathways associated with synaptic plasticity and neuronal survival 24. Oligomerization and polymerization processes form dimers, trimers, and other intricate oligomers, which exhibit enhanced stability and bioactivity compared to monomeric structures 25. The substitution patterns on stilbenes' phenyl rings substantially influence their spatial orientation and electrical properties. Consequently, this impacts their interaction with target proteins and receptors 26.

Stilbenes exhibit many neuroprotective mechanisms, including antioxidant characteristics, anti-inflammatory effects, modulation of signaling pathways, and the capacity to hinder the production of amyloid proteins 27. They collect and remove free radicals and reactive oxygen species (ROS), therefore reducing oxidative damage to brain cells and preserving mitochondrial function. They impede the production of pro-inflammatory cytokines, such as TNF-α and IL-1β, and limit the brain's activation of microglia and astrocytes 28. They regulate crucial communication pathways, enhancing neuroplasticity and optimizing synaptic transmission between neurons 29. Furthermore, they impede the aggregation and sedimentation of misfolded proteins, such as Aβ and tau, which are distinctive characteristics of Alzheimer's disease (AD) and other NDs. Lastly, they identify specific molecular markers associated with the compound's efficacy and disease progression, enabling personalized treatment approaches and patient selection in clinical trials 30. Biomarker research endeavors aim to identify distinct biological patterns associated with the reaction to stilbene and the progression of illnesses 31. Subsequently, this information may be used to create customized treatment strategies and categorize patients in clinical studies. In general, stilbenes are a class of chemical compounds that show significant promise for treating NDs. Continual study is underway to comprehend and harness their medicinal capabilities 32.

## 3. Stilbenes in neurological disorders

### 3.1 Alzheimer's disease

**Figure 2:**
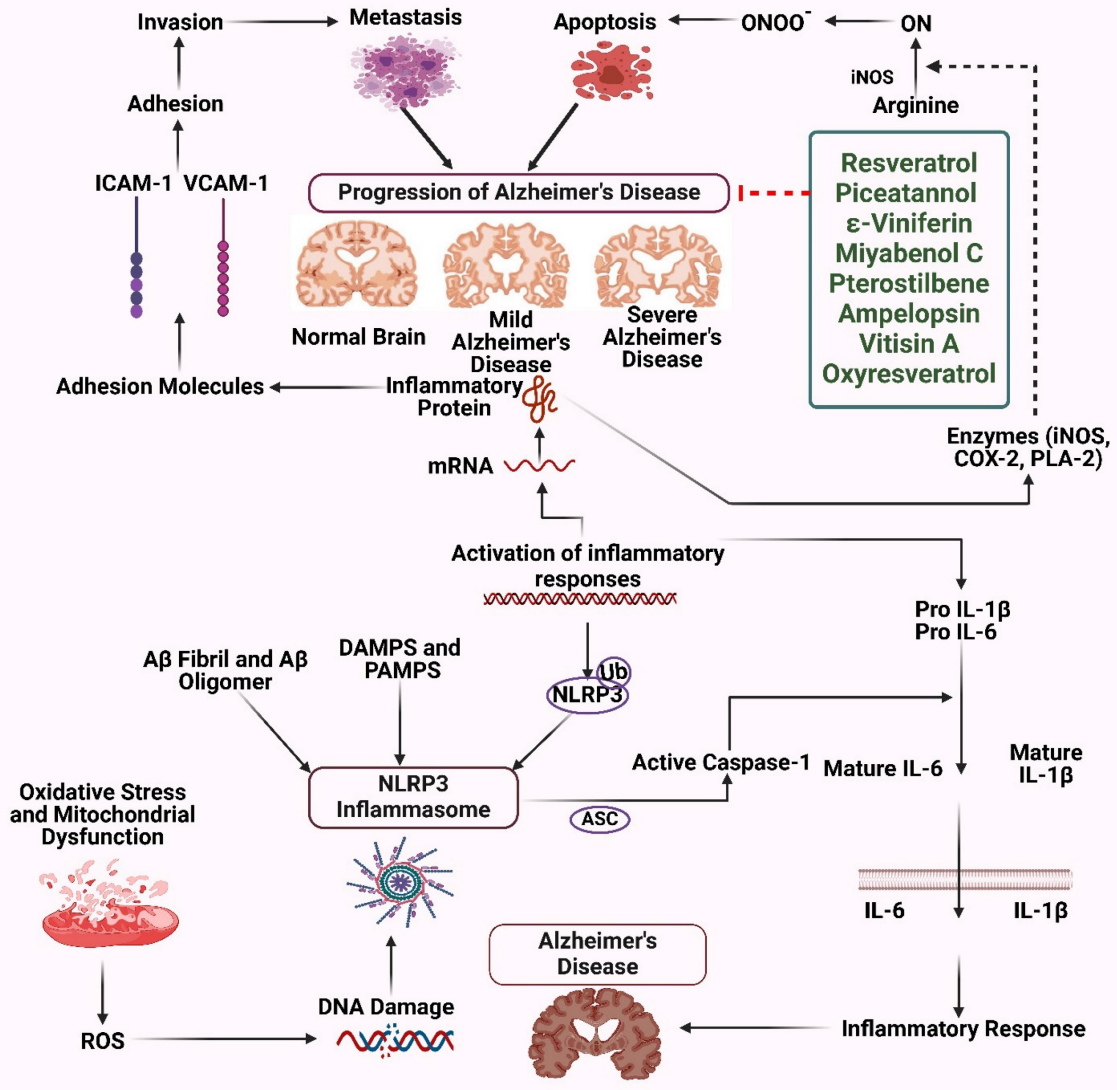
Stilbenes as therapeutics for Alzheimer's disease. The figure was designed by the Biorender.com program (https://biorender.com/, accessed on 1 May 2024).

AD is a widespread condition characterized by the progressive degeneration of the nervous system, and it is believed that a new instance arises every three seconds. The illness has a worldwide impact on 50 million individuals and is projected to increase significantly to reach 152 million people by the year 2050. The primary histological abnormalities seen in AD are the presence of neurofibrillary tangles (NFTs) and senile plaques inside the brain. These abrasions are formed due to the aggregation of Aβ peptides and the accumulation of tau protein that is hyperphosphorylated within neurons. The genes responsible for rare familial AD are mutated 33. Numerous experimental reports have focused on the Anti-Alzheimer potential of stilbenes. For instance, Monisha *et al.* conducted a study to examine the impact of trans-resveratrol on brain function and oxidative damage in rats provoked by streptozotocin. The animals were administered streptozotocin and subjected to a 21-day treatment with trans-resveratrol. The findings demonstrated that trans-resveratrol effectively mitigated cognitive decline and oxidative damage, suggesting its potential therapeutic use in the treatment of neurological conditions such as AD. The research emphasizes the possible benefits of trans-resveratrol in mitigating cognitive impairments and OS 34. Resveratrol has shown neuroprotective properties in animal research, namely in rat models. Modest consumption of red wine may effectively decrease amyloid-AD neurological pathology and alleviate memory impairment in mice. Research indicates that accumulating soluble Aβ oligomers outside of cells is a primary factor contributing to AD cognition and memory impairment development. Administration of polyphenolic chemicals derived from grape seed extract can potentially decrease cognitive decline in rats (Figure 2) 35. Administration of resveratrol in mice decreases the presence of stimulated microglia and inflammatory conditions, even if it affects the accumulation of amyloid. Research conducted on a mice model indicates that the administration of resveratrol to high-grade mice throughout ambulatory locomotive work results in a reduction in rearing behavior. Resveratrol enhances the activity of mitochondria in muscular and fatty tissues, resulting in improved aerobic efficacy, energy expenditure, and sensorimotor performance 36. A research investigation examining the impact of resveratrol on the brains of mice has shown that providing resveratrol supplements for 10 months safeguards against memory decline and neurological dysfunction in mice. Additionally, it enhances memory and thinking skills in normal mice. Resveratrol also decreases anxiousness and the accumulation of Aβ and p-tau clumps in the brain's hippocampus of mice. The research further discovered elevated quantities of the amyloid-breaking enzyme neprilysin, decreased BACE1 activity, and higher ribosomal protein levels. Resveratrol further stimulates AMPK, increasing the expression of SIRT1 and CREB. This implies that it may improve cognitive function and offer neurological protection in contrast to tau protein and Aβ-related diseases 37. Resveratrol enhances the activity of SIRT1 by increasing the stability of interactions between proteins and substrates. Research indicates that the administration of resveratrol alters the N-terminal region of SIRT1, resulting in enhanced affinity between the substrate and SIRT1. The compound enhances the levels of SIRT1 mRNA expression while mitigating any negative impacts on SIRT1 activities. Resveratrol can decrease apoptosis, hinder the body's inflammatory response, decrease damage caused by free radicals, and encourage the restoration of self-digestion in human neuroblastoma cells via the SIRT1 pathway. Research has shown that resveratrol has a role in preserving PC12 Aβ25-35 cells by increasing the activity of SIRT1 and preventing damage caused by microglia-associated Aβ toxicity via inhibiting nuclear factor kappa B (NF-κB) signaling. These results indicate that resveratrol can provide neuroprotection against AD by enhancing the activity of SIRT1 38,39.

In both *in vitro* and animal trials, hydroxylated stilbenes, which may be identified in fruits and medicinal plants, were investigated to determine their impact on parameters related to AD. In terms of the scavenging of DPPH radicals and suppressive actions against ACETYL CH and Aβ protein clumps, it was discovered that rhapontigenin (RHA), piceatannol, and isorhapontigenin (isoRHA) exhibited the highest levels of activity. During passive avoidance tests, mice given piceatannol demonstrated increased learning behavior. On the other hand, RHA and isoRHA promoted neurite outgrowths in SH-SY5Y cellular models. Based on these findings, it seems that nutritional supplements that include piceatannol may have the potential to be used for the amelioration of NDs 40. Aline *et al.* discovered that viniferin can reduce amyloid plaques and neuroinflammation in transgenic mice with the APPswePS1dE9 mutation. The mice were administered 20 mg/kg of viniferin, resveratrol, or its carrier, PEG 200. The cognitive condition of the mice was evaluated using the MWM test. The findings indicated that viniferin had a greater efficacy in reducing hippocampus amyloid loads and deposits than resveratrol. Additionally, both therapies offered partial protection against cognitive deterioration. Viniferin also reduced the amount of the [18F]DPA-714 taken up by the brain compared to resveratrol. Nevertheless, the neuroprotective efficacy of viniferin was obscured by the presence of PEG 200, a substance that induces neuroinflammation 41.

Further research was conducted to assess the comparative efficacy of pterostilbene and resveratrol in enhancing functional impairments and disease progression of AD in mice models. The investigators discovered that administering pterostilbene resulted in a considerable enhancement in arm water maze performance in mice compared to animals that were not given pterostilbene. Neither resveratrol nor pterostilbene showed an upsurge in sirtuin 1 expression or downstream indicators of sirtuin 1 stimulation. Nevertheless, pterostilbene effectively regulated stress indicators in cells, inflammatory processes, and AD pathology, linked to an increase in the expression of PPAR alpha. The results indicate that pterostilbene can influence cognition and cell stress more than resveratrol when given at the same dosages that food may achieve 42. The research examines the effects of Ampelopsin on proinflammatory cytokines, OS-related products, and 8-OHdG in the hippocampus of rats with AD. The findings indicate that AMP reduces the increase of pro-inflammatory cytokines (PICs) and the production of OS while inhibiting NOX4 in the hippocampus of rats with AD. Ampelopsin enhances cognitive function in rats with AD via the activation of signaling pathways associated with neuroinflammation and oxidative damage. The study's findings indicate that Ampelopsin notably enhances memory impairment in AD rats by suppressing neuroinflammation and triggering OS pathways. This suggests that Ampelopsin has the potential to serve as a supplemental intervention for preventing and alleviating cognitive deficits in AD 43. Oxyresveratrol has been discovered to enhance autophagy signaling and reduce the generation of APP in primary cortical astrocytes. This was confirmed using immunofluorescence and immunoblotting assays. Oxyresveratrol enhanced the autophagy flow, decreased the autophagy substrate p62/SQSTM1 amounts, and elevated p62 levels. Preconditioning with chloroquine and oxyresveratrol resulted in increased levels of LC3-II protein and counts of LC3 puncta, suggesting that oxyresveratrol-induced autophagy relied on the class III PI3-kinase pathway. Preconditioning with compound C, an inhibitor of AMPK, led to reduced production of LC3-II protein. The administration of Oxyresveratrol significantly increased the expression of ULK1, suggesting that ULK1 played a crucial role in starting autophagy induced by oxyresveratrol. Nevertheless, the administration of oxyresveratrol resulted in an upregulation of the expression of LAMP1. The work demonstrates that oxyresveratrol controls the initiation of autophagy and the decrease of APP in mouse cortical astrocytes via the regulation of the AMPK /mTOR pathway 44.

### 3.2 Parkinson's disease

Parkinson's disease (PD) is an ND defined by the presence of Lewy pathology and the gradual decline of neurons that produce dopamine in the substantia nigra. The intricate nature of the condition presents therapeutic difficulties and a lack of effective therapies. Resveratrol, a naturally occurring polyphenol molecule, has been shown to have neuroprotective benefits on many NDs. Resveratrol therapy effectively reduced motor and cognitive disorders in the PD mouse model in a way that depended on the dosage. The therapeutic benefits of resveratrol in treating PD are attributed to its ability to suppress the formation of aggregates of α-synuclein, decrease the levels of oligomers and total α-synuclein, reduce neuroinflammation, and alleviate OS. The results indicate that resveratrol has promise as a therapy option for PD and other disorders characterized by the accumulation of synuclein proteins 45. Gaballah *et al.* conducted a study to examine the impact of resveratrol on the regulation of ER stress-induced apoptosis, inflammatory responses, and OS indicators in the PD rat model. The scientists assessed the mRNA expression level of ER stress indicators, such as CHOP and GRP78, in the brains of rats. In addition, they evaluated the functioning of caspase-3, levels of IL-1β, and the DNA-binding ability of Nrf2. The findings demonstrated that resveratrol improved the harmful effects of rotenone-mediated ER stress by reducing the expression of GRP8 and CHOP genes. It also inhibited caspase-3 activity, reestablished the equilibrium of redox reactions by preventing XO action and carbonyl protein development, and maintained cellular antioxidant levels by stimulating glutathione peroxidase and Nrf2 pathways. Resveratrol has been identified as a promising therapeutic drug for protecting the nervous system in PD 46.

**Figure 3:**
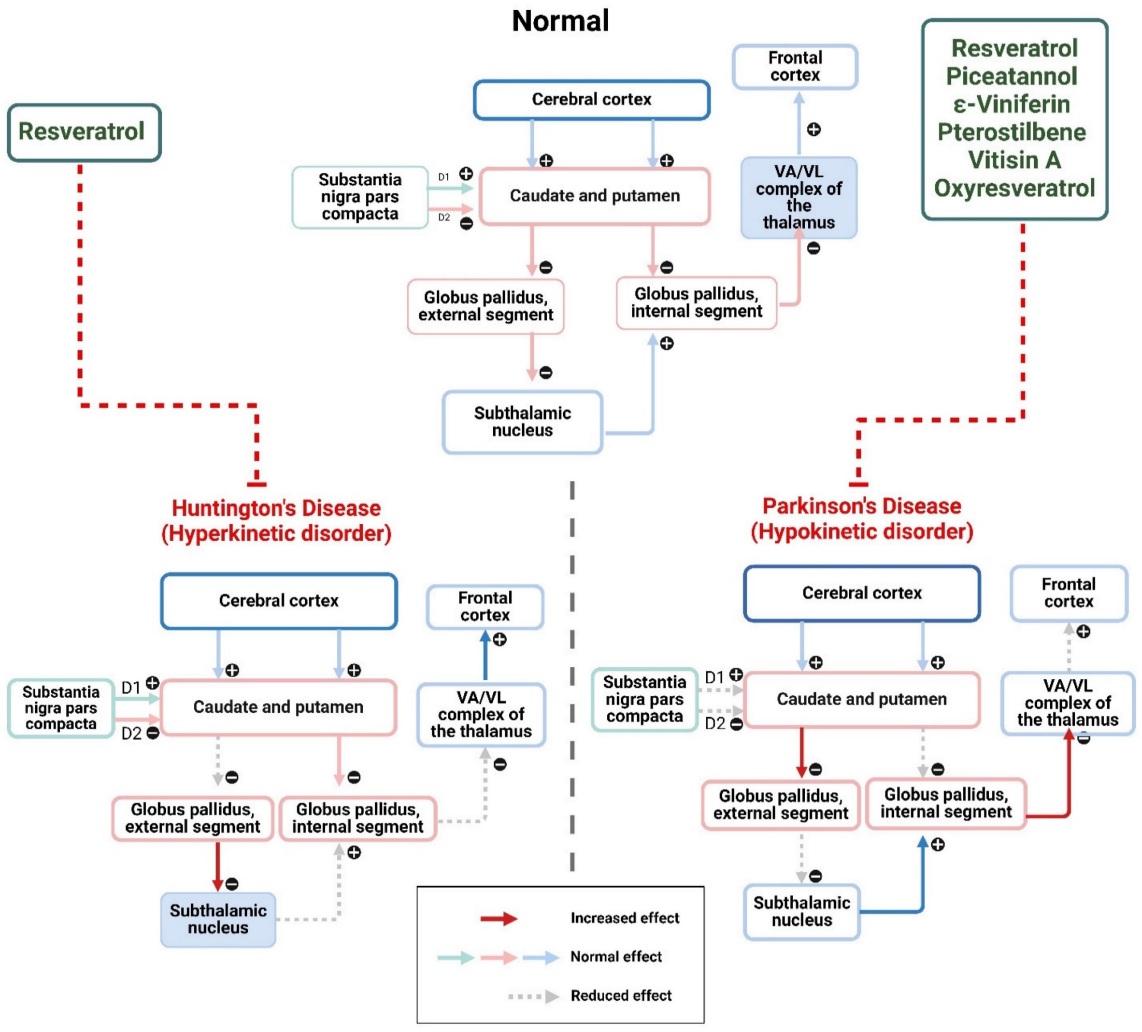
Stilbenes as therapeutics of Parkinson's disease and Huntington's disease. The figure was designed by the Biorender.com program (https://biorender.com/, accessed on 1 May 2024).

Resveratrol has demonstrated neurological protection against the impairment inflicted by MPTP, 6-OHDA, and rotenone *in vitro* experiments. These substances are believed to impact dopaminergic neurons. Research on mice revealed that resveratrol has neuroprotective effects towards MPTP, 6-OHDA, and rotenone. It effectively mitigates the cognitive and motor impairments caused by these neurotoxins. These results are corroborated by trials conducted on rats, providing more evidence that resveratrol might be a promising therapy for PD. Resveratrol has demonstrated promise as a therapy for PD, and PD offers opportunities for more studies and treatments (Figure 3) 47,48. Neurotoxins have been associated with harm by triggering apoptotic pathways. This stimulation leads to an upsurge in the ratio of Bcl-2 to Bax, reducing the release of cytochrome C and the activation of caspase-3. Resveratrol has been associated with its ability to protect the nervous system by acting as an antioxidant. It does this by reducing the generation of ROS and enhancing the body's defenses against antioxidants when exposed to rotenone. These results are corroborated by research demonstrating that resveratrol protects against mitochondrial dysfunction caused by experimental PD. It counteracts mitochondrial structure and electrical potential alterations while promoting new mitochondria generation. In summary, research has shown that resveratrol's antioxidant effects are advantageous in decreasing apoptosis and preserving mitochondria function 49. Sara and her team conducted a study examining piceatannol's defensive impact on peripheral nerve damage in animals. A pre-injection conditioning period of 7 days was provided before a single injection of cisplatin, followed by biochemical, behavioral, and histological tests. The findings indicated that the cisplatin infusion reduced sensitivity to heat, decreased ability to move, weakened grip strength, and lowered neurotensin levels. Additionally, piceatannol was shown to repair microscopic changes in the axons of nerves and reestablish the average myelin thickness, indicating that piceatannol may have a protective effect on peripheral nerve damage caused by cisplatin 50. Pterostilbene was discovered to shield SH-SY5Y cells from inflammatory reactions and damage in a coculture system, including SH-SY5Y neuroblastoma and BV-2 microglia. Pterostilbene enhanced the structure, survival, and LDH release, reducing programmed cell death and OS. Additionally, it increased the SIRT-1 expression and inhibited the acetylation of the NF-κB p65, reducing inflammatory markers such as L-6 and TNF-α. Nevertheless, the impact of these effects was counteracted by EX527. According to the research, pterostilbene has decreased the inflammatory response caused by microglia and diminished neuronal death and oxidative damage 51. Sergi and his team conducted a study to evaluate the neuroprotective and anti-inflammatory effects of viniferin in PC12 cells, which are used as a model for PD. Before injecting 6-OHDA, a compound that causes parkinsonism in rats, the neuronal cells were subjected to pre-treatment with viniferin, resveratrol, or a combination of both. The research further investigated the potential of viniferin, resveratrol, or their combination to mitigate LPS-caused inflammation in microglia. The findings indicate that viniferin and a combination of viniferin and resveratrol protect neuronal dopaminergic cells, shielding them from the harmful effects of 6-OHDA-caused apoptosis and cytotoxicity. Viniferin, when applied as a pre-treatment for microglia cells, decreased the harmful of glial stimulation on neurons 52. Oxyresveratrol, a substance that protects the nervous system, has been demonstrated to shield cells from damage caused by OS and apoptosis in a laboratory model of Parkinson's disease. Anuri *et al.* examined if this neuroprotective mechanism also applies to ER stress related to PD. The findings demonstrated that oxyresveratrol provides different forms of protection. The 6-OHDA model suppresses the gene expression of transcription factor 4, which regulates the destiny of pro-apoptotic protein. The α-syn model inhibited the production of mutant A30P oligomers, decreasing the ER-chaperone, 78-kDa Grp78. These findings indicate that oxyresveratrol is suitable for treating multifactorial diseases such as PD 53. Rodsiri *et al.* researched to examine the neuroprotective properties of oxyresveratrol on rotenone-induced parkinsonism. The OXY rats were orally administered oxyresveratrol from days 1 to 20, whereas rotenone was administered subcutaneously on days 15 to 20. The rotarod test was used to assess motor function. The brains were examined to determine the presence of dopaminergic neurons, levels of MDA, and the activities of SOD and catalase. The findings demonstrated that prior administration of oxyresveratrol improves the motor dysfunction caused by rotenone and protects the dopaminergic neurons. Oxyresveratrol's neuroprotective action is associated with its antioxidant capabilities 54.

### 3.3 Huntington disease

Huntington's disease (HD) is a hereditary ND that leads to problems with movement, mental health, and cognitive abilities. The condition is linked to an erratic increase in the CAG sequence in the huntingtin gene, with afflicted people having more than 40 sets of three nucleotides. The initiation and intensity of the disease are strongly linked to the quantity of CAG repeats, while the purpose of the trinucleotide sequence is still unclear 55. Sirtuins are enzymes dependent on NAD crucial in regulating various biological processes such as cell survival, transcription, and metabolism. SIRT1, a sirtuin found in mammals, has been linked to extending longevity and promoting neuronal survival. One of its targets is PGC-1α, a pivotal regulator of energy metabolism. In the case of HD, researchers have explored the effects of activating SIRT1 using resveratrol, a compound known to activate SIRT1, specifically in a transgenic mouse model. The study found that the administration of resveratrol, known as SRT501-M, led to an increase in the expression of PGC-1α in brown adipose tissue. However, this effect was not observed in the striatum, where NRF-1, PGC-1α, and the mitochondrial transcription factor remained unaffected. Furthermore, SRT501-M administration resulted in a reduction in vacuolation in BAT and a decrease in elevated blood glucose levels. Nevertheless, despite these positive effects, the treatment did not significantly improve motor performance, weight loss, survival rates, or striatal atrophy in the mice. In summary, while activating the PGC-1α signaling pathway through resveratrol-induced SIRT1 activation appears to be a practical therapeutic approach in brown adipose tissue, its efficacy in the central nervous system of HD transgenic mice remains limited 56. Naia *et al.* conducted a study to examine the effects of resveratrol and nicotinamide in mitigating mitochondrial damage in a model of HD. High-definition cell models exhibited a decrease in MMP and respiration, suggesting a deterioration in the function of the mitochondria. Resveratrol reinstated these variables, but nicotinamide elevated NAD^+^ levels, resulting in a beneficial enhancement of mitochondrial activity *in vitro* models of HD. Resveratrol reduced the level of H3 acetylation, while NAM enhanced the level of H3 acetylation. Administration with resveratrol for a continuous period of 28 days resulted in enhanced motor integration and learning. Conversely, nicotinamide hindered the transcription process connected to mitochondria, hence exacerbating the motor phenotype. The research proposes that the increase of deacetylase action by resveratrol may have a partial role in regulating motor abnormalities associated with HD 57.

### 3.4 Cerebral ischemia

Cerebral ischemia (CI) is a significant worldwide health problem, and it is projected that the expenditures associated with stroke will amount to $240.67 billion by 2030. Existing therapies, such as anti-thrombolytics and hypothermia, have not substantially decreased neuronal damage, neurological impairments, and death rates. There is a pressing need for new and innovative treatments 58. Lei *et al.* conducted a study to examine resveratrol's neurotherapeutic properties on brain injuries, inflammatory conditions, and rupture of the blood brain barrier (BBB) in animals with localized CI. Intraperitoneal administration of resveratrol with 10 and 100 mg/kg was performed on SD rats after ischemia. The research discovered that resveratrol substantially reduced neurological impairment scores, infarct dimensions, damaged neurons, MPO action, and EB contents. The levels of expression of NF-κB p65, TLR4, COX-2, TNF-α, MMP-9, and IL-1β increased due to ischemia, whereas resveratrol decreased levels of these factors. According to the research, resveratrol has been shown to reduce inflammation, BBB interruption, and brain injury in rats after experiencing localized CI 59. Dou *et al.* discovered that applying resveratrol shortly after the commencement of artery occlusion resulted in a promotion of Th1/Th2 equilibrium. At 3 days after the ischemic event, it was shown that the expression of inflammatory cytokines in the small intestine was reduced due to the modulation of the intestinal flora. Resveratrol reduced the small intestine's enhanced permeability caused by CI, reducing the intestines' pro-inflammatory immune response and lowering permeability. It effectively prevented the breakdown of the BBB in the striatum and caused inflammation after a stroke, three days after the stroke occurred. Furthermore, it reduced the occurrence of small brain infarcts and minimized neurological impairments by reducing the levels of cytokines in the infarct region three days after a stroke. This research shows that resveratrol might potentially prevent inflammation after a stroke by influencing the balance of certain immune cells in the intestines 60. Shan *et al.* examine the efficacy of resveratrol in treating neurological inflammation caused by CI. The study used an animal model created by blocking the cerebral artery and administering resveratrol by IP injection over multiple intervals after an ischemic stroke. The levels of miR-155 and the characteristic genes of microglia in the damaged brain were quantified using ELISA and RT-PCR. Resveratrol's impact on inflammation in LPS-stimulated BV2 microglia was investigated using tests *in vitro*. The findings indicated that resveratrol facilitated the M2 polarization and diminished neural inflammation in the damaged brain while stimulating BV2 microglia. Resveratrol can decrease neurological inflammation shortly after brain ischemia by reducing miR-155 61. An extensive review of 564 research revealed that the administration of resveratrol resulted in a considerable reduction in the extent of tissue damage caused by stroke and improved the neurological function of rats, as measured by their neurobehavioral scoring, compared with a control group. The research employed a randomized model and a 10-item questionnaire to evaluate the quality ratings. The findings indicated that sub-groups administered with resveratrol at a dose range of 20-50 mg/kg exhibited substantial reductions in infarct and neurobehavioral scoring volumes. The research demonstrates that administering resveratrol can offer neuroprotective benefits in models of ischemic stroke. This finding can potentially inform forthcoming clinical studies, which might substantially impact human health. The discoveries might guide further preclinical and clinical experiments 62. CI results in augmented OS, an issue that may be alleviated by the phenolic molecule resveratrol, known for its anti-inflammatory and anti-oxidant features. The study conducted by Ro *et al.* aims to determine whether the neuroprotective action of resveratrol during CI is linked to its ability to regulate the levels of trace elements and hazardous metals. The findings demonstrated that rats administered resveratrol before the experiment exhibited elevated levels of trace elements and enhanced antioxidant function in the cerebral cortex compared to rats that did not receive any treatment. In addition, prior therapy with resveratrol reduced lipid peroxidation levels and hazardous metal concentrations. The research proposes that resveratrol's neuroprotective impact is achieved by controlling the amounts of trace elements (lead), peroxidation of lipids, and antioxidant function in the brain 63. The administration of resveratrol at the onset of reperfusion led to an increase in the levels of p-AMPK in the cerebral cortex, which was subjected to glutamate-associated excitotoxicity. The survival rate of rats given MCAO was raised as a result. The administration of resveratrol in nerve cells resulted in a decrease in the formation of superoxide anion, the prevention of intracellular Ca^2+^ overload, a reduction in the release of LDH enzyme, and a reduction in mortality. In addition, it encouraged mitophagy. However, the impact of resveratrol was hindered by the suppression of AMPK stimulation with compound C, which suggests that the neurotherapeutic action of resveratrol is partly dependent on its stimulation of the autophagy/AMPK pathway 64.

Neonatal hypoxic-ischemic encephalopathy, often known as HIE, is a severe condition that manifests itself in the form of cognitive decline. The creation of novel medications that may alleviate the cognitive impairment caused by HIE is of the utmost importance. It has been discovered that resveratrol, a SIRT1 agonist, has a potent anti-inflammatory impact; however, its effect on poor synaptic plasticity in HIE is yet unknown. In mice with HIE, resveratrol was used in research that showed that it improved prolonged cognitive and memory impairments, decreased the amount of damage to hippocampal neurons, and increased spine density and synaptic expression of proteins. The SIRT1/NF-κB axis may play a role in regulating this effect. As a result of this work, a novel conceptual basis for using resveratrol to avert prolonged neurological damage after HIE has been established 65. Yan and his team conducted a study to examine the neuroprotective effects of pterostilbene in mouse models with CI caused by MCAO. The rats were categorized into groups: sham, normal, MCAO, and MCAO plus pterostilbene (25 mg/kg). The rats were orally administered pterostilbene for 30 days, after which they underwent MCAO initiation. The findings demonstrated that administering pterostilbene led to substantial enhancements in body weight, suppression of glucose levels, reduction in renal, hepatic, and cardiac characteristics, drop in glutathione levels, and downregulation of cytokines associated with inflammation and parameters. Additionally, it inhibited cell swelling, collapse, and infiltration of macrophages and monocytes, as well as the degranulation of polymorphonuclear leukocytes in the brain. Pterostilbene had a neuroprotective impact on CI in rats via its anti-inflammatory pathway 66. Wang *et al.* conducted a study to look into the action of piceatannol on enhancing cognitive functioning in cerebral ischemia/reperfusion injury (CIRI). The mice were administered either a small or high amount of Pic for one hour following oral CIRI administration, which was repeated once daily for 6 days. The research discovered that Pic enhanced cognitive abilities and alleviated hippocampus neuronal pathology after CIRI. Pic at both low and high dosages reduced ROS formation and the protein expression linked to apoptosis while simultaneously increasing the antioxidant enzyme levels. The administration of pic therapy also stimulated the Sirt1 pathway, resulting in an elevation in the proportion of TUNEL-(+)ve cells and an upregulation of CC-3 expression. The inhibitory impact of Sirt1 on Pic's antioxidant properties was seen both *in vitro* and *in vivo* 67. Wang *et al.* conducted a study to evaluate the antioxidant activities of pic in CIRI. The study included an *in vitro* model (oxygen-glucose depletion) and an *in vivo* model (MCAO). An analysis was conducted on the impact of piceatannol on histological results and the inner structure of the cortex. The findings indicated that cell survival was enhanced in the piceatannol groups, whereas the levels of SOD, GPOx, LDH, and MDA were reduced. The neurologic severity scoring and infarct volume in the piceatannol groups exhibited a reduction compared to the model group. Piceatannol partly reduced the observed damage on histological testing. Piceatannol dramatically upregulated the expression of HO-1, Nrf2, and NQO1. The research indicated that piceatannol had a defensive impact against CIRI 68.

### 3.5 Anxiety and depression

Anxiety and depressive disorders have a modest level of heritability, with a common genetic risk factor that is shared with internalizing illnesses. Neuroticism, a characteristic of one's personality, is linked to both diseases. Non-hereditary risk factors that are often seen include traumatic experiences, parenting practices, and exposure to stress. Emotion-regulatory mechanisms are mediated by changes in neurological circuits within prefrontal-limbic pathways. Anxiety disorders and severe depression often manifest throughout different stages of development. Anxiety disorders generally arise during preadolescence and early adolescence, whereas major depression tends to emerge during adolescence and early to mid-adulthood. Research indicates that anxiety problems often occur before the onset of severe depressive illness. Early childhood behavioral inhibition or a very anxious temperament might heighten the probability of developing a social anxiety disorder, thereby increasing the vulnerability to severe depressive illness and drug dependence 138. Zadeh and colleagues conducted a study to explore the potential neuroprotective properties of resveratrol in mitigating arsenic-associated neurotoxicity in rats. The experiment involved the categorization of rats into seven distinct groups: control, vehicle, arsenic (As) alone, as treated with resveratrol at doses of 10 mg/kg and 20 mg/kg, resveratrol alone at doses of 10 mg/kg and 20 mg/kg. The cognitive impairment and molecular alterations induced by As were evaluated through behavioral assessments and biomarker analyses. The findings revealed a pronounced impairment in cognitive performance among rats exposed to arsenic. However, the administration of resveratrol at a dose of 20 mg/kg demonstrated significant improvements in various behavioral parameters.

Furthermore, resveratrol at this dose mitigated molecular changes associated with arsenic toxicity, such as enhancing antioxidant capacity, restoring glutathione levels, and reducing malondialdehyde levels. Additionally, resveratrol at a dose of 10 mg/kg was observed to elevate FRAP and GSH levels but did not significantly affect MDA levels. Overall, the behavioral outcomes suggest a neuroprotective role for resveratrol against arsenic-induced toxicity 110. Yuan *et al.* conducted a study examining the impact of trans-resveratrol on anxiety and neuropathic pain triggered by stress in mice. Employing a well-established animal model to simulate stress disorder, the research yielded noteworthy outcomes. The findings reveal that trans-resveratrol effectively mitigates stress-associated anxiety and alleviates pain responses in mice without significantly affecting locomotion distance. Furthermore, the study observed a stress-mediated cold and mechanical allodynia reversal following trans-resveratrol administration. Notably, trans-resveratrol counteracted abnormalities in the limbic hypothalamus-pituitary-adrenal axis, restoring the differential expression of glucocorticoid receptors in regions associated with anxiety and pain. Additionally, it elevated protein kinase A levels, p- cAMP protein, and BDNF, which were diminished in mice subjected to stress. These findings suggest that trans-resveratrol exhibits neuroprotective properties by safeguarding neurons against stress-caused damage. This protective mechanism involves the stimulation of downstream neuroprotective molecules 114. Finnell and colleagues examined the efficacy of resveratrol in mitigating chronic stress-caused inflammation in animals subjected to social exclusion. Rats engaged in proactive coping exhibited more upright postures and extended delay periods before experiencing defeat. In contrast, rats that engaged in passive coping had elevated levels of inflammatory proteins in the brain area associated with depression that is involved in the release of noradrenaline. Resveratrol at a dosage of 30mg/kg effectively prevented neurological inflammation. Resveratrol decreased the expression of cytokines in the dorsal raphe (DR) without impacting the activity of serotonin. The results indicate that passive coping rats may exhibit depressive behavior as a result of social stress, which is likely caused by neurological inflammation triggered by the stress 117.

A preclinical investigation explored the impact of resveratrol on mood fluctuations in rats subjected to SIS. The rats were segregated into six groups: a control group, a SIS+NS group, a fluoxetine group, and three resveratrol+NS groups. Behavioral assessments unveiled that exposure to SIS induced feelings of depression and anxiety, with the most pronounced effects observed in the SIS+NS group. Notably, resveratrol demonstrated potential as an antidepressant in comparison to fluoxetine. The administration of resveratrol at varying doses (20-80 mg/kg) ameliorated depression and anxiety symptoms in rats subjected to chronic SIS. These findings suggest that resveratrol could serve as both an antidepressant and anxiolytic agent, thereby offering promising therapeutic potential 118. Research conducted on mice used a model including the removal of both ovaries and the simulation of hormones to evaluate behavior resembling postpartum depression. Resveratrol evaluated mice's SIRT1 levels, autophagy indicators, and mTOR pathway. The findings suggested that resveratrol enhanced postpartum depression by increasing the activity of SIRT1 and autophagy indicators, reducing the p62 protein expression, and suppressing the activation of AKT and mTOR via inhibition in the hippocampus. Resveratrol can potentially reduce postpartum depression in mice by activating SIRT1, enhancing autophagy, and suppressing the mTOR pathway 119. The research examined the impact of resveratrol on behavior in the hippocampus of rats subjected to persistent social isolation (SI) stress. The animals were administered resveratrol (20-80 mg/kg) for 28 days. Behavioral tests indicated that SI resulted in an upsurge in immobility, a rise in blood corticosterone levels, and a decrease in hippocampus SOD and CAT activities. The administration of resveratrol had a considerable positive effect on behavioral changes via regulating neurological inflammation and OS. However, the dosage of 20 mg/kg of resveratrol was ineffective in addressing depressive behavior generated by SI. The results indicate that resveratrol reduced depressive behavior in mice exposed to extended stress, perhaps via enhancing blood corticosterone levels and improving hippocampus inflammation and OS indicators 120. A research investigation explored the impact of pterostilbene on the pentetrazol (PTZ)--cause kindling model of epilepsy in mice, aiming to assess its potential neuroprotective and anticonvulsant properties. Throughout the study, mice underwent repeated administrations of pterostilbene across a dosage range of 50-200 mg/kg. The effects of this treatment on various parameters, including anxiety and depression, were carefully examined. Findings indicated a significant reduction in seizure activity with a pterostilbene dosage of 200 mg/kg. However, there were no observable alterations in locomotor action or depressive symptoms. Notably, pterostilbene intervention reversed the decline in GABA concentration caused by PTZ kindling. Conversely, mRNA expression levels of IL-6, GABRA1A, IL-1β, and GRIN2B remained unaffected by the pterostilbene treatment 123.

### 3.6 Autism spectrum disorder

Autism spectrum disorder (ASD) is impacted by both environmental and genetic factors, including exposure to valproic acid during pregnancy. ASD is a health condition that is marked by difficulties in social communication, repetitive habits, limited interests, and sensory problems. The worldwide prevalence of this condition is less than 1%; however, it is more common in high-income nations. While it is not classified as hereditary, post-mortem examinations, neuroimaging, and electrophysiological tests reveal minor variations. Psychosocial interventions can potentially enhance behaviors; however, further study is necessary to ascertain the enduring requirements and therapies 139. A study examined the impact of administering resveratrol during pregnancy on social interactions in a mouse autism model. The results indicated a decrease in site choice and no particular choice between roaming a wire cage or a wire cage. Administering resveratrol therapy throughout the prenatal stage successfully averted these social deficits. A computational approach was employed to eliminate molecular connections between resveratrol and valproic acid, uncovering a feeble and volatile interaction power and indicating potential impacts on cells. This work proposes a potentially effective method to investigate novel molecular targets associated with the cause of autism and the developmental changes related to neurological and behavioral deficits in individuals with ASD 140. Fontes *et al.* studied the impact of resveratrol exposure on sensory behavior, neuron distribution, and excitatory/inhibitory synapse proteins in an autism model. Resveratrol was administered regularly to pregnant rats, and valproic acid was injected into them at the E12.5 stage. The findings revealed modified PV+-neurons in the primary sensory cortex and the hippocampus, decreased levels of gephyrin in the somatosensory region, and no changes induced by valproic acid. The administration of resveratrol averted these modifications. The research emphasizes the significance of sensory elements in ASD and the interaction between resveratrol and the valproic acid model in examining the underlying mechanisms of ASD 126. Ranjana *et al.* conducted a study to explore the capacity of resveratrol to decrease neurological inflammation in an ASD rat model. The rats were subjected to IV injection of a 1M concentration of Propanoic acid to elicit symptoms similar to ASD, and resveratrol was supplied for 4 weeks. The animals underwent assessments for several behavioral contexts and biochemical testing to evaluate OS, mitochondrial complex systems, and MMP-9. The findings demonstrated that the administration of resveratrol effectively and in a manner that, depending on the dosage, reversed all impairments in behavioral and biochemical aspects associated with the autistic characteristics produced by propanoic acid. The research found that resveratrol effectively reversed the main symptoms of autism, including those related to OS, impaired mitochondrial function, MMP-9, and TNF-α expression. This was shown in rats with autism produced by PPA. Thus, resveratrol has the potential to be used as an additional treatment drug to improve neurobehavioral and biochemical impairments related to ASD 127.

Chemokine receptors have a vital function in the CNS and are implicated in developing neuroinflammatory disorders. Resveratrol, a commonly prescribed medication for the treatment of NDs, has undergone a thorough investigation into its connection to autism. Research was done on mice to investigate the impact of these animals on the chemokine receptors C-C and C-X-C motifs in CD4+ T cells. The findings indicated that BTBR animals had elevated CCR and CXCR synthesis and transcription levels in CD4+ T cells compared to control mice. Resveratrol therapy reduced the synthesis and CCR and CXCR expression in CD4+ T cells compared to the control groups that did not receive treatment. In addition, in brain tissues, the administration of resveratrol resulted in a reduction in the mRNA expression level of CCR and CXCR. These findings indicate that resveratrol reduced the amounts of chemokine receptors, which might serve as specific targets in prospective autism treatments 128. Iohanna *et al.* assessed brain water content, BBB permeability, aquaporin 1 and 4 expression, and GFAP in a valproic acid-rat model. The rats demonstrated higher permeability to dye and elevated volume of brain water after 1 month, but resveratrol prohibited this. Valproic acid has been observed to lead to a decline in aquaporin 1 across various brain regions, a decrease in aquaporin 4 within the cortex, and an elevation in aquaporin 4 levels within the somatosensory area. Moreover, resveratrol has been shown to augment the population of astrocytes and enhance GFAP fluorescence. These findings underscore the potential of resveratrol in advancing our understanding of the underlying etiology and mechanisms involved in ASD 130.

### 3.7 Multiple sclerosis

**Figure 4:**
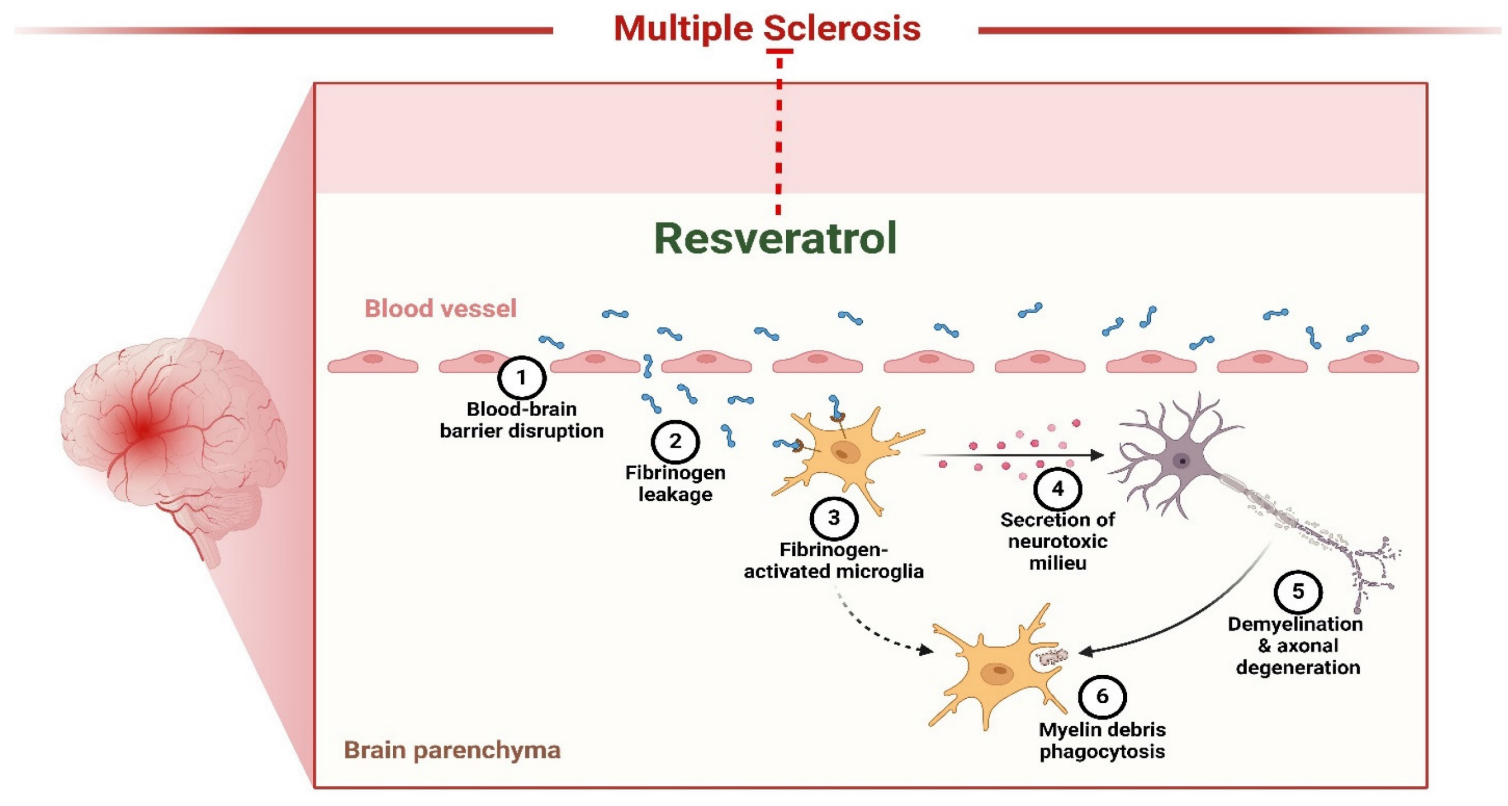
Resveratrol as therapeutics of Multiple Sclerosis. The figure was designed by the Biorender.com program (https://biorender.com/, accessed on 5 May 2024).

Multiple sclerosis (MS) is a neurological disorder in which the immune system targets and damages the myelin, a protective covering of the brain and spinal cord. This damage disrupts the communication between the brain and other body parts 141. Resveratrol inhibits neuronal degeneration in mice afflicted with recurrent encephalomyelitis, an example of MS. However, reports indicate it may inhibit inflammation in persistent encephalomyelitis (Figure 4). However, its neurotherapeutic benefits have not yet been assessed. Recent research has investigated resveratrol's possible neuroprotective and immune-modulating properties in chronic encephalomyelitis produced by vaccination with glycoprotein peptides in mice. The findings demonstrated that resveratrol effectively postponed the initiation of encephalomyelitis but did not modify the characteristics of inflammation in spinal cords. Nevertheless, it had notable neuroprotective properties, as shown by an increased abundance of retinal ganglion cells in the mice eyes with neuroinflammation that received treatment with resveratrol during encephalomyelitis. These data indicate that resveratrol might provide additional advantages when used with anti-inflammatory therapies for MS 134. Heba *et al.* examined the pro-remyelination benefits of resveratrol in mice affected with cuprizone. The mice were given a food containing 0.7% cuprizone for 7 days, after which they were switched to a diet containing 0.2% cuprizone for 3 weeks. The administration of Resveratrol commenced during the subsequent week and continued for 3 weeks. The brain contents and functions of cyt were evaluated. Oxidase and SOD were measured. Researchers discovered that resveratrol restored motor balance and coordination, repaired demyelination, boosted mitochondrial activity, relieved OS, and reduced NF-κB activation. Furthermore, it led to an augmentation in the expression of Olig1, a factor that is favorably associated with the process of active remyelination. This implies that resveratrol could benefit the remyelination process, making it more effective in treating MS 136. A separate investigation discovered that miRNA generated by resveratrol reduced the severity of encephalomyelitis, a mouse model used to research MS. Resveratrol treatment reduced the extent of the condition, including the levels of inflammation and the invasion of immune cells. The miRNA microarray study revealed a modified miRNA profile in encephalitogenic mice subjected to resveratrol therapy. Resveratrol influenced the course of the cell phase and the occurrence of apoptosis in activated T cells, particularly in the brain. The administration of resveratrol during encephalomyelitis significantly increased the expression of miR-124 while simultaneously inhibiting the expression of the target gene, SK1. These data indicate that therapy with resveratrol improves the development of encephalomyelitis by suppressing neurological inflammation by modifying the miR-124/SK1 axis, stopping the advancement of the cell cycle, and increasing death in stimulated encephalitogenic T cells 137.

## 4. Clinical status

Resveratrol is advantageous in treating AD in both *in vitro* and *in vivo* experiments. Nevertheless, there has not been a comprehensive clinical study conducted on a wide scale so far, and the limited capacity of resveratrol to be absorbed by the body and dissolve in water presents challenges in detecting unaltered resveratrol in the bloodstream. Although there is evidence from experiments conducted on living organisms, there is still a shortage of conclusive outcomes from trials involving humans 35,142. Scientists are investigating strategies to effectively treat resveratrol, with some studies demonstrating the same qualities that protect the nervous system. Current clinical studies are being conducted to examine the impact of this treatment on neurological disorders such as AD. Resveratrol derivatives with enhanced bioavailability, effectiveness, and stability are now undergoing testing to assess their potential in treating several degenerative disorders. Resveratrol derivatives have been shown to provide neuroprotective properties in both *in vivo* and *in vitro* models, as indicated by many research 35,49. Although there is a limited amount of clinical research on the impact of resveratrol in AD, two phase 2 clinical trials have demonstrated that resveratrol is effective for individuals with mild to severe AD and modifies many AD biomarkers. In the experiments conducted by Muossa and Turner, patients were administered resveratrol for 1 year 143,144. Patients who received resveratrol treatment had reduced levels of MMP-9, which is responsible for breaking down components of the extracellular matrix. This activity is linked to AD and neurodegeneration. In contrast, patients in the placebo group did not see the same drop in MMP-9 levels. The reduction in MMP-9 suggests that resveratrol strengthens the central nervous system by decreasing its permeability, limiting the access of pro-inflammatory substances to the brain. In addition, those who were administered resveratrol saw a decelerated decrease in the levels of Aβ42 and Aβ40 in their CSF, suggesting a reduced accumulation of Aβ in the brain 145. While being rapidly digested and having limited bioavailability, resveratrol has been discovered in large concentrations in the CSF, showing its potential to penetrate the BBB in several experiments effectively 143,144. Both clinical trials yielded results consistent with those from *in vitro* and *in vivo* research. They offered data supporting the potential of resveratrol as a secure and productive therapy for AD 146.

Research was conducted to assess the effectiveness of resveratrol supplementation in improving cognitive function in patients diagnosed with schizophrenia. The research included 19 male participants ranging in age from 18 to 65. The study design was a randomized and double-blind experiment. The individuals were allocated to either a group receiving resveratrol supplementation (200 mg) or a group receiving a placebo (200 mg). Neuropsychological performance and degree of psychopathology were evaluated using cognitive tests. The findings indicated that there were no notable improvements in neurological function or reduction in schizophrenia intensity after one month of resveratrol intake. The research found that adding resveratrol did not restore memory 147. Zhu *et al.* conducted a study to assess the tolerability, safety, and effectiveness of an oral formulation, including resveratrol, in reducing the AD development rate. A total of thirty-nine participants diagnosed with moderate to severe AD underwent screening for this research. The results revealed that the treatment group had a reduced rate of decline compared to the control group after 12 months of therapy. However, no scores achieved statistical significance. Falls were the most prevalent adverse event seen in the control group. The study's findings indicate that administering low-dose resveratrol is effective and tolerated well. However, more research is required to provide more comprehensive insights 148. An experiment was conducted to evaluate the therapeutic benefits of combining resveratrol on irritability in individuals with ASD. The study was double-blind, meaning neither the participants nor the researchers knew which treatment they were receiving. Additionally, the trial was placebo-controlled, meaning some participants received a placebo instead of the treatment. The findings indicated that resveratrol did not have a noteworthy impact on irritation or ratings related to hyperactivity. Nevertheless, the group that received resveratrol had a more significant decrease in scores related to non-compliance and hyperactivity. The experiment yielded the first findings suggesting that resveratrol may enhance hyperactivity/non-compliance in individuals with ASD 149.

## 5. Concluding remarks and future directions

Stilbenes have displayed encouraging neuroprotective properties in preclinical investigations, providing potential for the treatment of disorders such as AD, PD, ischemic stroke, and so on. These chemicals possess anti-inflammatory, antioxidant, anti-amyloidogenic, and neurogenic characteristics, indicating their ability to address multiple pathogenic pathways contributing to NDs. Moving from preclinical potential to clinical use presents difficulties because it requires meticulous preparation and implementation. This includes establishing suitable dosages, comprehending how drugs are absorbed, distributed, metabolized, and excreted in human subjects, and evaluating possible interactions with other medications. Due to the diverse nature of NDs, it is necessary to use specific therapies and personalized healthcare strategies. To advance the use of stilbenes in clinical settings, more research should focus on understanding the specific pathways through which stilbenes provide neuroprotective benefits. Nanotechnology-based delivery systems should also be explored to enhance stilbene availability and tissue targeting. Conducting extensive clinical studies is essential for assessing the safety and effectiveness of stilbenes in various human populations. These trials should include many patients and apply rigorous research methods. Longitudinal studies are necessary to evaluate the prolonged impacts of stilbene therapy and its ability to alter disease development. The transition of stilbenes from preclinical data to clinical applications has the potential to significantly improve neurological therapies, offering hope and better standards of life for patients with NDs.

## Figures and Tables

**Table 1:** Preclinical findings regarding the use of stilbenes in neurological disorders.

Disease name	Compound name	Study model	Dose/conc.	Findings	Ref.
Alzheimer's disease	Resveratrol	Mice	40 mg/kg	Resveratrol reversed Aβ associated memory deficits and controlled neuroinflammation.	(69)
		Mice	10 and 20 mg/kg	Resveratrol inhibited the Aβ associated microglial stimulation.	(34)
		Transgenic mice	100 mg/kg	Improved cognitive functionality and offered neuroprotection against Aβ and tau pathologies.	(37)
		Mice	0.02 mg/kg	Treatment with resveratrol substantially alleviated biochemical and behavioral abnormalities caused by Aβ1-42.	(70)
		AβPP/PS1 mice	16 mg/kg	Resveratrol facilitated alterations in inflammatory processes and prohibited memory loss.	(71)
		Tg19959 mice	300 mg/kg	Resveratrol exhibited a region-specific reduction in the development of plaque.	(72)
		Transgenic 5XFAD mice	0.1%	Improved the detrimental effect on the brain caused by HFD.	(73)
		HT22 cells	0.1-20 µM	Treatment with resveratrol prevented AMPK from being phosphorylated.	(74)
		PC12 cells	20 μM	Resveratrol and other small compounds with comparable conformational selectivity helped identify the epitopes causing Aβ-associated toxicity.	(75)
		C57BL/6J mice	200 mg/kg	Resveratrol reduced peripheral and cerebral inflammation, metabolic abnormalities, and memory loss in mice.	(36)
	Piceatannol	SH-SY5Y cells	10-50 μM	Piceatannol outperformed AChE suppressive actions and anti-Aβ self-aggregations.	(40)
		PC-12 cells	5-40 μM	Piceatannol reduced oxidative stress and mitochondrial-associated apoptosis in PC-12 cells, resulting in cytoprotection.	(76)
		bEnd.3 cells	10-50 μM	Piceatannol decreased cerebral cell endothelial oxidative stress and inflammation *in vitro*.	(77)
	ε-Viniferin	Transgenic mice	20 mg/kg	Viniferin decreased brain absorption of [18F]DPA-714 more than resveratrol.	(41)
		Transgenic mice	10 mg/kg	Trans ε-viniferin lowered amyloid size and density and reduced microglia and astrocyte reactivity.	(78)
	Miyabenol C	APP/PS1 mice	0.6 μg/g	Although miyabenol C did not change the protein levels of the β-secretase BACE1, it inhibited its activity *in vivo*.	(79)
	Pterostilbene	SAMP8 mice	120 mg/kg	Pterostilbene potentially modulated cognition and cellular stress, possibly due to increased PPAR alpha expression and increased lipophilicity.	(42)
	Ampelopsin	Rats	-	Ampelopsin substantially amended memory deficit in AD rats by preventing neuroinflammation.	(43)
		C57BL/6 mice	10 ng/µL	Ampelopsin A improved intrinsic neuronal excitability and neuroprotection.	(80)
	Vitisin A	ICR mice	0.25-4 μM	Vitisin A decreased scopolamine-induced AChE activity and MDA levels and boosted brain BDNF expressions.	(81)
	Oxyresveratrol	Mouse cortical astrocytes	10 µM	CORT-mediated APP synthesis was dramatically reduced by oxyresveratrol via autophagy.	(44)
		BV-2 Cells, Mice	25 μM, 50-100 mg/kg	Oxyresveratrol reduced neuroinflammation caused by LPS, improving cognition and episodic memory.	(82)
Parkinson's disease	Resveratrol	SNCA mice	50 mg/kg	Ameliorated cognitive deficit and reduced neuroinflammation and improved motor functionality	(45)
		C57BL/6 mice	100 mg/kg	Ameliorated cognitive deficit, enhanced iron content, improved motor functionality	(48)
		Wistar albino rats	20 mg/kg	Resveratrol decreased brain caspase-3 activity and gene expression in rats to diminish ER stress.	(46)
		Wistar rats	40 mg/kg	Decreased mitochondria dysfunction, reduced oxidative damage, and improved cognitive functioning.	(83)
		Mice	50 mg/kg	Improved motor functionality, increased neuronal survival, suppressed neural apoptosis	(84)
		SD rats	15 and 30 mg/kg	Resveratrol-activated PI3K/Akt to impede 6-OHDA-mediated apoptosis.	(85)
		Balb/c mice	10 mg/kg	Resveratrol treated group showed lower IL-1β levels and enhanced pAkt/Akt ratio, helping dopamine neuron survival.	(86)
		Wistar rats	2.5 and 10 mg/kg	Resveratrol maintained dopamine levels.	(87)
		SK-N-SH and SH-SY5Y cells	25-100 μM	RES mediated the SNHG1/miR-128-3p/SNCA axis to promote cell autophagy and inhibit PD development.	(88)
		mutant *D. melanogaster*	0-60 mg/kg	Resveratrol decreased parkin dysfunctional mutation-related oxidative stress and locomotor impairment.	(89)
	Piceatannol	Rats	10 mg/kg	Piceatannol mostly repaired microscopical changes in nerve axons and recovered typical myelin thickness.	(50)
		Rats	-	Post-SAH hippocampal apoptosis, cellular edema, and pyknosis were dramatically decreased.	(90)
	Piceatannol-3′-O-β-D-glucopyranoside	N2a cells, male mice	3.91-125 μM	Reduced nerve cell apoptosis caused by oxidative stress and damage to mitochondria.	(91)
	Pterostilbene	BV-2 and SH-SY5Y cells	2.5-10 μM	Pterostilbene decreased neuronal apoptosis, oxidative damage, and inflammation.	(51)
	Vitisin A	C57BL/6 mice	1-10 μM	BDNF-CREB signaling upregulation by vitisin A partly protects neurons.	(92)
	ε-Viniferin	PC12 cells	10^-9^ M	Viniferin inhibited glial-induced neuronal cytotoxicity.	(52)
	Oxyresveratrol	Mes23.5 cells	0-50 μM	The 6-OHDA model was protected by oxyresveratrol, which inhibited ATF4 transcription.	(53)
		SH-SY5Y cells	20 μM	Oxyresveratrol treatment raised 7,8-dihydrobiopterin levels, mostly from tyrosine, phenylalanine, and tryptophan metabolism.	(93)
		Wistar rats	300 mg/kg	Oxyresveratrol treatment protected dopaminergic neurons and decreased rotenone-induced motor impairment.	(54)
Cerebral ischemia	Resveratrol	Rats	10 and 100 mg/kg	Resveratrol decreased BBB disruption, inflammation, and brain injury	(59)
		C57BL/6 mice	200 mg/kg	Resveratrol prevented post-stroke BBB disturbance, decreased brain infarcts, and lowered neurological impairments.	(60)
		Mice	30 mg/kg	SIRT1 stimulation may explain resveratrol postconditioning's neuroprotective effects.	(94)
		Rats	30 mg/kg	Resveratrol administration lowered neurological impairment scores, improved neuronal stem survival, and prevented microglia and astrocyte stimulation.	(95)
		Wistar female rats	0.15 mg/kg	Resveratrol administration effectively decreased hypoxic-ischemic offspring brain damage.	(96)
		C57/BL mice	100 mg/kg	Resveratrol inhibited miR-155 to increase microglia M2 polarization and reduce neurological inflammation after cerebral ischemia.	(61)
		SD rats	10-30 mg/kg	Resveratrol improved neurological performance, infarct area, and cortical ischemia in brain infarction rats.	(97)
		SD rats	30 mg/kg	Resveratrol mitigated neuronal apoptosis in brain ischemia caused by MCAO.	(98)
		Wistar rats	1.9 mg/kg	Resveratrol lowered infarct size, cerebral edema, and BBB impairment, improving neurological function and survival rate.	(99)
		Rats	20 mg/kg	Resveratrol administration substantially decreased lipid peroxidation and hazardous Pb levels in cerebral ischemia rats.	(63)
		Rats	1.8 mg/Kg	Resveratrol decreased infarct size and improved MCAO-treated rat mortality.	(64)
		Mice	-	Resveratrol improved hippocampus neuronal structure, dendritic spine density, and synaptic protein expression.	(65)
		SD Rats	10 mg/kg	Resveratrol restored mitochondrial metabolism, improving hcNPC therapeutic effectiveness and neural differentiation.	(100)
		Wistar rats	1.9 mg/kg	Resveratrol protects neurons from ischemia-associated injury and inhibits GLUT3 overexpression in brains.	(101)
		Wistar rats	60 mg/kg	Resveratrol reduces oxidative damage, ER stress, and neurological inflammation to prevent subarachnoid hemorrhage.	(102)
		Mice	-	RES reduced the CD147/MMP-9 axis to suppress pro-inflammatory microglia, preventing ischemic brain damage.	(103)
		SD rats	20 mg/kg	Resveratrol inhibited transition elements, lipid peroxidation, and toxic metals to prevent brain ischemia damage.	(104)
		Rats	10 mg/kg	Resveratrol downregulated the ERK pathway to decrease IR-induced neuronal death in cerebral hemorrhage models.	(105)
		Rats	-	Resveratrol administration reduced ferroptosis in neurons.	(106)
		C57Bl/6J mice	-	Resveratrol improves memory and protects memory-processing brain regions.	(107)
	Pterostilbene	Rats	25 mg/kg	Pterostilbene's anti-inflammatory activity protected rats from brain ischemia.	(66)
		SD rats	7-28 mg/kg	Pterostilbene reduced neuronal scores, brain-H_2_O content, and infarction volume.	(108)
		C57BL/6 mice	10 mg/kg and 20 mg/kg	Pterostilbene protected hippocampus neurons via Sirt1/FoxO1.	(67)
		ICR mice, PC12 cell	2.5-40 μM	Piceatannol dramatically upregulated HO-1, Nrf2, and NQO1.	(68)
	Piceatannol	Rats	1-10 mg/kg	Piceatannol reduced p-JNK3 and neuronal death during ischemia.	(109)
Anxiety and depression	Resveratrol	Rats	10 and 20 mg/kg	Resveratrol reduced arsenic-related learning and memory impairments in rats.	(110)
		Wistar rats	20 mg/kg	Resveratrol lowered anxiety, enhanced burrowing behavior, and decreased spatial memory.	(111)
		Mice	40 mg/kg	Resveratrol reduced arsenic-associated cognitive impairment.	(112)
		ICR mice	2.5-10 mg/kg	Resveratrol inhibited PDE4D and activated the cAMP pathway, offering antidepressant effects.	(113)
		ICR mice	10-40 mg/kg	Resveratrol provided neuroprotection against posttraumatic stress disorder.	(114)
		Wistar rats	10-40 mg/kg	Resveratrol regulated 5-HT-dependent signaling to strengthen brain functioning.	(115)
		SH-SY5Y cells	10-25 μM	Resveratrol increased the viability of the cells.	(116)
		SD rats	10, 30 mg/kg	Resveratrol reduced neuroinflammation.	(117)
		Wistar rats	20-80 mg/kg	Resveratrol reduced rat feelings of depression and anxiety.	(118)
		C57BL/6 mice	-	Resveratrol reduced depressive actions in mice via activating SIRT1 and autophagy.	(119)
		Wistar rats	20-80 mg/kg	Resveratrol mitigated depressive responses in animals under stress.	(120)
		Wistar rats	80 mg/kg	Resveratrol possessed potent antidepressant properties.	(121)
	Pterostilbene	Swiss mice	50-200 mg/kg	Combining carbamazepine with pterostilbene reduced muscle strength but not motor coordination.	(122)
		Mice	50-200 mg/kg	Pterostilbene decreased seizure events in mice but not locomotion activity or symptoms of anxiety.	(123)
	Piceatannol	Mice	1-30 mg/kg	Low doses are responsible for the anxiolytic effect	(124)
Autism spectrum disorder	Resveratrol	Wistar rats	3.6 mg/kg	Resveratrol prevented autistic behavior.	(125)
		Wistar rats	3.6 mg/kg	Increased accurate choice percentage.	(126)
		SD rats	5-15 mg/kg	Resveratrol restored socializing, stereotyping, locomotion, memory, and depression.	(127)
		BTBR model	20-40 mg/kg	Repetition behavior is reduced.	(128)
		C57BL6/J mice	30 mg/kg	Resveratrol enhanced social interaction in adult ASD mice after single dosing.	(129)
		Wistar rats	300 mg/kg	Resveratrol reduced cerebral edema and BBB permeability in ASD animal models.	(130)
		Wistar rats	3.6 mg/Kg	Resveratrol has been shown to prevent long-term hippocampus changes and regulate interneuron architecture in ASD.	(131)
		Wistar rats	3.6 mg/Kg	In pups who received VPA during pregnancy, resveratrol avoided developmental delays.	(132)
		Wistar rats	3.6 mg/kg	Resveratrol mitigated cytoarchitectural and interneuronal alterations.	(133)
Multiple Sclerosis	Resveratrol	C57/Bl6 mice	250 mg/kg	Resveratrol protects against neuronal death in persistent demyelinating sickness.	(134)
		SJL/J mice	500 mg/kg	Oral SRT501 effectively mitigated the loss of neurons in cases of ocular neuritis.	(135)
		C57Bl/6 mice	250 mg/kg	Resveratrol enhanced motor coordination, reversed demyelination, enhanced mitochondrial activity, reduced oxidative stress, and suppressed NF-κB activation.	(136)
		Mice	100 mg/kg	Resveratrol therapy elevated miR-124, decreasing SK1, suggesting immunological regulation.	(137)
Huntington disease	Resveratrol	Transgenic mice	25 mg/mouse/day	Resveratrol has been shown to protect against peripheral impairments in HD models.	(56)
		YAC128 mice	1 and 5 μM	Resveratrol increased mitochondrial transcription of genes in HD, presumably controlling motor abnormalities.	(57)

**Table 2:** Clinical findings regarding the use of resveratrol in neurological disorders.

Patients	Dose/conc.	Findings	Ref.
80	150 mg/day	Improved cognition and mood status.	(150)
22	250 and 500 mg	Blood flow increased dose-dependently in the cerebral region.	(151)
119	1000 mg/day	No considerable findings were observed.	(144)
30	25 mg	Reduced depression and anxiety	(152)
46	200 mg/day	Memory performance improved a lot.	(153)
19	200 mg/day	Didn't improve cognitive function.	(147)
29	5 mg	Low-dose is secure and tolerated well, while its impact on clinical outcomes remains unknown.	(148)
64	500 mg/day	Loss of brain volume was restored.	(143)
31	500 mg/day	Found no significant impact.	(149)
125	150 mg/day	Relieve chronic pain in osteoarthritis associated with aging and improve the overall well-being of women after menopause.	(154)
